# Randomised feasibility trial and embedded qualitative process evaluation of a new intervention to facilitate the involvement of older patients with multimorbidity in decision-making about their healthcare during general practice consultations: the VOLITION study protocol

**DOI:** 10.1186/s40814-020-00699-7

**Published:** 2020-10-26

**Authors:** Joanne Butterworth, Suzanne Richards, Fiona Warren, Emma Pitchforth, John Campbell

**Affiliations:** 1 Exeter Collaboration for Academic Primary Care (APEx), University of Exeter Medical School, Room 110, Smeall building, St Luke’s campus, Magdalen Road, Exeter, EX1 2LU UK; 2 Leeds Institute of Health Sciences, School of Medicine, University of Leeds, Leeds, UK

**Keywords:** Primary care, Elderly, Older people, Multimorbidity, Shared decision-making, Patient involvement

## Abstract

**Background:**

The number of older people with multiple health problems is increasing worldwide. This creates a strain on clinicians and the health service when delivering clinical care to this patient group, who themselves carry a large treatment burden. Despite shared decision-making being acknowledged by healthcare organisations as a priority feature of clinical care, older patients with multimorbidity are less often involved in decision-making when compared with younger patients, with some evidence suggesting associated health inequalities. Interventions aimed at facilitating shared decision-making between doctors and patients are outdated in their assessments of today’s older patient population who need support in prioritising complex care needs in order to maximise quality of life and day-to-day function.

**Aims:**

- To undertake feasibility testing of an intervention (‘VOLITION’) aimed at facilitating the involvement of older patients with more than one long-term health problem in shared decision-making about their healthcare during GP consultations.
- To inform the design of a fully powered trial to assess intervention effectiveness.

**Methods:**

This study is a cluster randomised controlled feasibility trial with qualitative process evaluation interviews. Participants are patients, aged 65 years and above with more than one long-term health problem (multimorbidity), and the GPs that they consult with. This study aims to recruit 6 GP practices, 18 GPs and 180 patients. The intervention comprises two components: (i) a half-day training workshop for GPs in shared decision-making; and (ii) a leaflet for patients that facilitate their engagement with shared decision-making. Intervention implementation will take 2 weeks (to complete delivery of both patient and GP components), and follow-up duration will be 12 weeks (from index consultation and commencement of data collection to final case note review and process evaluation interview). The trial will run from 01/01/20 to 31/01/21; 1 year 31 days.

**Discussion:**

Shared decision-making for older people with multimorbidity in general practice is under-researched. Emerging clinical guidelines advise a patient-centred approach, to reduce treatment burden and focus on quality of life alongside disease control. The systematic development, testing and evaluation of an intervention is warranted and timely. This study will test the feasibility of implementing a new intervention in UK general practice for future evaluation as a part of routine care.

**Trial registration:**

CLINICAL TRIALS.GOV registration number NCT03786315, registered 24/12/18

## Background

It is estimated that by 2032, there will be over 13.5 million patients in the UK aged 65 years and over, an increase of 50% when compared with 9 million in 2012 [36]. The prevalence of multimorbidity (more than one long-term health condition) increases with age [1]; 58% in people over 60 years compared with 14% in those under 40 years [30, 42, 43]. In developed countries, the prevalence of multimorbidity is also higher in females, among those with lower socioeconomic status (SES) and in minority ethnic groups [11, 45]. In those with lower SES, it is more common for mental and physical illness to co-exist. In addition, patients’ experiences of multimorbidity are affected by psychosocial and behavioural factors and larger health inequalities have been observed in vulnerable groups [39].

Consequences of ageing with multimorbidity include functional decline with poor quality of life and high healthcare costs [1, 26, 32]. The Department of Health [15] reported that older patients (those aged 65 and above) consult increasingly frequently (12–14 times/year in 2008/2009 vs. 6–7 times in 1995). Healthcare practitioners are often ‘medicalising’ the care of older people, diagnosing and treating each condition separately with new medication for example, and there are increasing rates of polypharmacy and new diagnoses [16].

There is evidence that involvement in healthcare decision-making is valued by older patients with multimorbidity [4, 9]. It is a means of effectively sharing the burden of treatment and enabling a holistic approach to clinical care [48]. Despite older patients typically expressing high levels of satisfaction with the care that they receive [7], they have been shown to be less frequently involved in decision-making when compared with younger, healthier patients [49], and there is a lack of recent evidence in this area. Therefore, whilst this patient group are often in receipt of ‘good-quality care’, there are concerns that this care is not currently meeting the complex needs of older patients with multimorbidity [48]. In addition, there is some evidence of an association between a lack of involvement and health inequalities for this patient group, for example, reduced rates of referral for postmenopausal bleeding and for hip pain [17, 28, 46].

Interventions aimed at facilitating shared decision-making between doctors and patients have previously focused on condition-specific clinical decisions. These interventions are therefore outdated in their assessments of today’s older patient population who often have complex needs as a result of multimorbidity [23, 38]. Recent literature suggests that new interventions are required in order to address older patients’ fundamental needs and life-goals, to support these patients in prioritising treatment options that maximise their quality of life and day-to-day function [48].

National Institute for Health and Care Excellence (NICE) guidance highlights the importance of delivering good-quality clinical care that takes account of multimorbidity [34]. The World Health Organisation [51] and UK health policy [14, 35] have identified shared decision-making as a priority feature of good-quality clinical care. Shared decision-making is a component of patient-centred care and is associated with patient adherence with treatment advice, satisfaction with healthcare and trust in the doctor [13, 20, 25, 37]. Policy makers require further evidence-based guidance regarding how to direct funding towards good quality clinical care for older patients with multimorbidity when consulting in general practice [5, 34].

An effective intervention in this area has the potential to address health inequalities through the facilitation of effective, personalised care that addresses patients’ fundamental needs holistically. The intervention has the potential to achieve positive outcomes for both patients and practitioners, along with reductions in primary healthcare costs for the National Health Service. This study aims (a) to feasibility test the delivery and evaluation of the intervention ‘VOLITION’, and (b) to carry out process evaluation of intervention and study procedures.

## Objectives

### Feasibility objectives

Feasibility objectives will assess:

- Capability to recruit and the resulting characteristics of the sample
- Data collection processes
- Potential outcome measures
- Resource and capacity for study management and intervention implementation
- Responses to the intervention by participants (tentative, preliminary evaluation)

### Process evaluation objectives

Process evaluation objectives will assess:

- Acceptability of the intervention to patients and GPs
- Acceptability of study procedures to practices and participants

The feasibility and process evaluation findings will together inform strategies to address any challenges in trial and intervention delivery. Findings will inform the design of a future fully powered, large-scale, randomised controlled trial to formally evaluate the effectiveness of the intervention.

## Methods

### Trial design

This study is a cluster randomised controlled feasibility trial with embedded qualitative process evaluation.

**Fig. 1:**
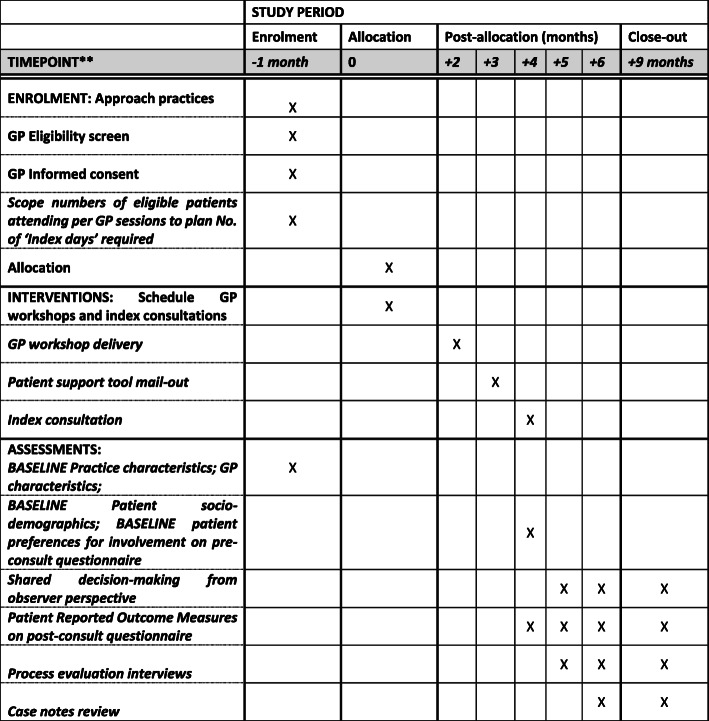
Testing VOLITION: schedule of enrolment, interventions and assessments

Figure 1 shows the schedule of enrolment, interventions and assessments as a SPIRIT figure.

### Sampling

Six GP practices will be recruited from rural Devon and from each of the cities of Exeter and Plymouth, UK. A minimum practice size of 3000 patients will ensure sufficient numbers of eligible participants. (It is anticipated that there will be approximately 800 older patients with multimorbidity in an average 8000 patient practice [1]). Table 1 shows the participant inclusion and exclusion criteria. An average of three (total = 18 GPs), and at least 2, GPs per practice will be recruited. Patients aged 65 and above with known multimorbidity, defined as the presence of two or more long-term conditions (as specified in Table 1), will be approached.

**Table 1:** Participant inclusion and exclusion criteria

Participant type	Inclusion criteria	Exclusion criteria
GPs	Only permanent GPs (partners and salaried) to avoid loss to follow-up.	Locum (sessional) GPs Trainee GPs (GPs will not be excluded based on less than full time working)
Patients	Patients aged 65 and above with known multimorbidity The condition should be one of^a^: angina or long-term heart problem; arthritis or long-term joint problem; asthma or long-term chest problem; blindness or severe visual impairment; cancer in the last five years; deafness or severe hearing impairment; diabetes; epilepsy; high blood pressure; kidney or liver disease; long-term back problem; long-term mental health problem; long-term neurological problem.	Temporary residents and vulnerable patients e.g. those recently bereaved, those with severe mental illness, severe cognitive impairment, end stage disease, communication difficulties e.g. physical impairment caused by a stroke as opposed to language barriers, a learning disability, or those unable to complete questionnaires or interviews for any other reason. (A minimum time for a condition to be ‘long-term’ will not be specified)

^a^This list was adapted from the English National General Practice Patient Survey [10]. Where dyads of conditions occur within the same organ system, e.g. anxiety and depression, these will only be counted once e.g. mental health problem

Patient participants will be identified using an automated practice database search strategy designed by the research team. The practice administrative team will use this ‘flag’ to identify ten consecutive potential participants with pre-booked consultations with each of the three recruited GPs. Random sampling of participants is not required as the clinical presentations, and therefore the nature of the decision-making between consecutive consultations, is expected to be random. A total of 180 (minimum 120) potential patients will be identified across 6 practices, with the aim of recruiting at least half.

The sample size is sufficient to compare between-group differences in quantitative feasibility objectives such as recruitment rates and data collection procedures including the completeness of participant questionnaires. This sample size also allows the estimation of standard deviations for the clinical outcome measures being tested. In addition, it is sufficient to obtain a purposively selected sub-sample of participants for process evaluation interviews, to achieve the qualitative feasibility objectives.

### Recruitment procedures

Non-compliance with the intervention will be taken into account at patient, GP or practice level by over-recruiting. Practices will receive email and phone correspondence inviting their participation. Practice recruitment will be dependent on an expression of interest from at least two of their GPs. Practices will be incentivised through payment for local coordination and set-up. GPs will receive a certificate for the training course (Williamson et al. 2007).

For each recruited GP, ten potential patient participants with pre-booked appointments will be screened by GPs, and if eligible, contacted by post ahead of their appointment. They will receive a cover letter, participant information sheet and example consent form. On their arrival in the practice waiting room, the receptionist will provide the patient with a pre-consultation questionnaire.

Patients who do not wish to participate will indicate this with a tick box on the pre-consultation questionnaire. Practices will keep an anonymised log of patients who declined to participate. A tick box on the post-consultation questionnaire will enable patients to opt out of process evaluation interviews and/or from having their case notes reviewed.

### Consent

Written consent from GPs will be obtained at the time of practice recruitment. Consent to identify and approach potential patient participants will be obtained from the practice manager. Written consent from patients will be obtained by the GP at the start of the consultation. GPs will be expressly clear that they are not part of the research team when taking consent. Written informed consent will be obtained prior to interviews for process evaluation purposes by the Chief Investigator. Where possible, reasons for participants declining participation will be recorded anonymously by the Chief Investigator or by GPs. Identifiable data will not be accessed without prior patient consent.

### Randomisation and blinding

The study will use a cluster randomised design. Clusters will be defined at practice level, with practices randomised 1:1 to usual care or to the intervention (90 patients per arm) following recruitment. Block randomisation will be stratified by site; two practices in inner city Plymouth, two practices from Exeter and two from rural Devon.

Randomisation will be conducted by an independent statistician who is blinded to the identity of practices. Blinding of the research team to outcome assessment will not be possible due to resource availability. Blinding of participants is unlikely to be achievable as participants in the intervention arm are likely to be aware that they are receiving more than standard ‘usual care’.

### The intervention

The overarching project ‘VOLITION’ follows the Intervention Mapping framework ([3]) as a means of systematically applying existing literature and relevant theory to each step of intervention development and evaluation. Social and behavioural science theory was used to describe factors affecting older patients’ involvement in decision-making about their healthcare, as well as methods for improvement. Evidence from a systematic review [8], from patient, public and expert opinion and from a focus group study with older patients with multimorbidity and with GPs, enabled the refinement of logic models underpinning this work. The Intervention Mapping process will be published separately.

**Fig. 2:**
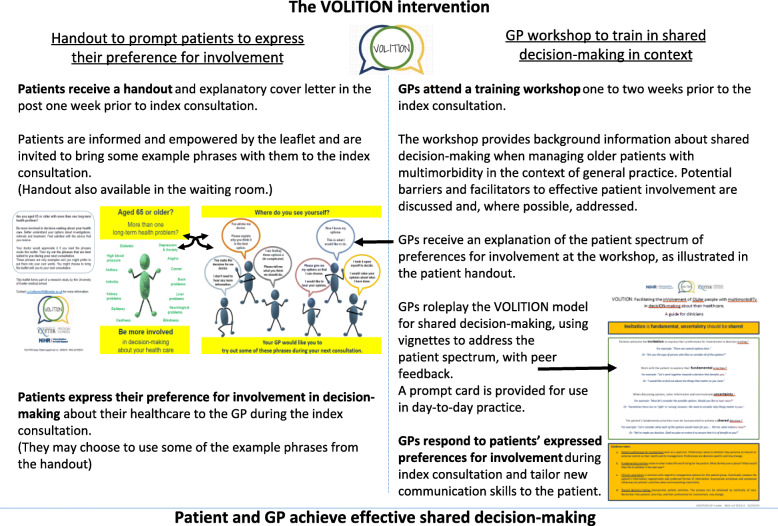
The VOLITION intervention

There are two core intervention components (see Table 2 and Fig. 2 for detail):

- A patient tool to facilitate patients to convey their preferences for involvement to the GP
- A workshop for GPs to train them in shared decision-making orientated communication skills, delivered in the context of the challenges currently facing GPs when consulting with this patient group

**Table 2:** The VOLITION intervention applications

Patient support tool^a^	GP workshop
**Central image**	**Delivery and facilitation by a GP**
Illustrates the spectrum of patient preferences for involvement and poses the question “where do you see yourself?”	For the purposes of role-modelling as a means of knowledge transfer
**Phrases to use during the consultation**	**Information provision**
Aimed at facilitating patients to ask for or decline participation in decision-making, accompany the central image.	GP-facilitator provides information regarding the elements of a shared decision-making approach to the consultation, in the context of older patients with multimorbidity.
Phrases are matched to the spectrum of patient preferences for involvement.	GP-facilitator delivers new messages about the potential benefits of shared decision-making. GP-facilitator uses a set of one or more meaningful premises and a conclusion to deliver these messages.
**Messages**	
Inform the patient of their right to ask for involvement in decision-making about their care.	GP-facilitator provides positive messages regarding the role of the patient and their preferences within shared decision-making. (This information is designed using evidence from the literature regarding GPs current beliefs around shared decision-making with this patient group.)
Suggest that the patient possesses the capability to state their preferences for involvement to the GP.	GP facilitator provides information about the importance and relevance of a patient-centred approach to the consultation.
Emphasise the benefits of shared decision-making for the patient.	Messages delivered by GP-facilitator suggest that the GP possesses the capability to use a shared decision-making approach to the consultation.
	GPs are asked to relay GP-facilitator’s messages to each other, and facilitator clarifies any confusion that appears during this process.
	**Role-play**
	GPs are first shown an example video-recorded consultation between the GP-facilitator (peer-model) and an actor-patient. GP-facilitator discusses the challenges of facilitating shared decision-making with the patient and how (s)he overcame them.
	GPs take turns as the GP, the patient and the observer to role-play clinical scenarios in threes.
	Individual GPs rehearse and repeat a shared decision-making approach to a role-play consultation, using a new ‘VOLITION’ model, incorporating a patient-centred, holistic approach.
	The role-play ‘patient’ states their preference for involvement in decision-making about their healthcare; their ideas, concerns and expectations; and their preferred data format for decision-related information. In this way the ‘patient’ prompts the GP to use appropriate communication skills to match their shared decision-making preferences.
	Clinical scenarios provide increasingly challenging tasks during role-play, with feedback from peers serving as an indicator of capability to the GP GP-facilitator encourages elaboration to augment the information provided in the crib sheet for the case scenario.
	**Reflective discussion in threes followed by group feedback**
	Discussion of GP’s appropriate response to patient preferences, fundamental priorities and requirements
	Discuss the experience and provide feedback to others.
	GP-facilitator encourages elaboration to augment the information provided in the crib sheet for the case scenario.
	**Supporting reference materials**
	Handbook containing the VOLITION model and all of the key messages delivered by the GP-facilitator during the workshop, the case scenarios for role-play and space for the individual to write reflective notes. Also available online.
	Online link to the video-recorded consultation between GP-facilitator and actor-patient.
	**Patient dialogue during index consultation**
	Patient provides a nudge to the GP in the form of a phrase from the patient support tool, informing the GP of their preferences for involvement in decision making about their care.
	Acts as a cue to the GP to adapt their communication skills accordingly.

^a^The support tool will also be displayed as a poster in the waiting room to prompt recall, as well as being available in leaflet form.

The patient tool will be mailed to eligible patients ahead of their index consultation. GPs will attend the training workshop no earlier than two weeks in advance of the index consultations with participating patients. Patients in the usual care group will receive standard care.

### Data collection

#### Practice and participant descriptive data

Practice level data (list size, location and deprivation, practice size, staffing, rural/urban) and GP participant data (age, gender, ethnicity and time since qualification) will be collected at the time of recruitment. Patient demographics (age, gender and ethnicity) and health status data will be collected via self-report using pre-consultation questionnaires.

#### Collecting outcome measure data

The General Practice Patient Survey (GPPS) uses five-point Likert scales [10]. A subset of these are appropriate for evaluation of Patient Reported Outcome Measures (PROMs) [13] on the pre-consultation patient questionnaires. These questionnaires will also be used to collect patients’ pre-consultation preferences for involvement in decision-making.

A post-consultation patient questionnaire will be used to collect post-consultation PROMs. Data will include the extent to which patients felt engaged in decision-making [2], their satisfaction with care and patient empowerment [27]. A post-consultation questionnaire for GPs will collect GPs’ ratings of the extent to which they felt they involved the patient in decision-making.

Video-recordings will enable the shared decision-making process to be assessed from an observer perspective using the OPTION(5) scale (Elwyn 2014). Two researchers will independently score each clinical decision made out of 100, with higher scores indicating better shared decision-making.

Case note review by two independent researchers will take place 12 weeks after the index consultation (to ensure time for documentation of the required outcomes). Information collected will include re-attendance within 28 days and referral for investigation or to secondary care.

### Analysis

#### Recruitment capability, consent and randomisation procedures, sample characteristics

The trial will determine whether it is possible to recruit appropriate study participants to test the intervention, or whether amendments are required ahead of a future trial. The flow of participants will be summarised in a Consort diagram. Numbers of eligible clusters, individual participants identified and randomised, recruitment rates and attrition will be recorded.

The search strategy used to identify potentially eligible patients will be tested on different practice databases with a view to finalising a generic, universal search algorithm. Anonymised data on ineligible patients will provide a profile of those frequently excluded. Consideration will be given to whether adaptations to the trial could be appropriate, to enable these patients to participate in a future study.

Any difficulties with meeting recruitment targets (6 practices; at least 2 GPs per practice; at least 5 patients per GP) will be documented. Field notes and correspondence with practices will enable the issues to be explored. Patients’ reasons for declining will be reviewed in light of recruitment procedures. For example, patients will use the pre-consultation questionnaire to indicate whether they received the information pack in the post. The extent to which over-recruitment is necessary will be reviewed, as will the importance of incentivising participants.

Randomisation procedures will be discussed at team meetings to inform amendments prior to a larger trial. For example, consideration will be given to randomising more practices to the intervention than to usual care, to gather more data regarding intervention implementation if required. Consideration will be given to initiating the study in each of the three sites sequentially (e.g. inner city, then urban, then rural) in order to increase trial management capacity. Consideration will be given to blinding the research team for data analysis in the future trial.

Rates of patient consent, to the use of questionnaire data, to video-recording ([12]; Asan and Montague 2014) and to case note review, will be evaluated. Numbers lost to follow-up will be reviewed; however, it is anticipated that this will be minimal. The study will determine whether there are differential drop-out or recruitment rates between intervention and control groups (indicative of selection bias).

The degree of sociodemographic diversity in the sample will be reported in respect of generalisability of results. The external validity of the sample will be reviewed by comparing to relevant external datasets when reporting recruitment, selection procedures and participation rates (Steckler and McLeroy 2007).

#### Evaluation of data collection

Table 3 outlines the feasibility measures to be assessed regarding the collection of outcome data.

**Table 3:** Evaluation of data collection procedures

Type of data collection (patient-, GP- or researcher-facing)	Components to be reviewed	Methods of analysis	Potential resultant amendments ahead of a definitive, larger trial
Patient-facing (questionnaires)		All patient-facing data collection forms have been reviewed for their suitability by Patient and Public Involvement (PPI) group	PPI members advised on the wording and format of questionnaires. PPI will provide a lay perspective on interpretation of feasibility findings.
	The feasibility of distribution and collection of patient questionnaires by receptionists.	Researcher’s field notes and follow-up correspondence with practices. Process evaluation interviews with patients.	Consideration of distribution by another means e.g. in the post (or email) prior to consultation.
	The appropriateness of collecting demographic data by patient self-report on pre-consultation questionnaires, as opposed to through the medical record.	Review of completeness of relevant items on patient questionnaires. Process evaluation interviews to discuss potential reasons for missing data. Correspondence with practices regarding practicalities of accessing patient demographic data with consent.	Consideration of accessing patient baseline data through practice records.
	Whether patient participants require assistance, encouragement or supervision to complete either of the questionnaires.	Researcher’s field notes and process evaluation interviews.	The provision of assistance to patients when completing questionnaires, e.g. providing staff to assist them or allowing more time pre- or post-consultation to allow the patient to seek help from a third party.
	Issues regarding time taken for questionnaire completion, any difficulties with comprehension of questionnaire items, numbers of questionnaires returned and reasons for missing data	Relevant quantitative data will be reported descriptively. Issues will be explored qualitatively with patients during process evaluation.	Time taken for completion may inform decisions surrounding when and how to distribute questionnaires. Issues regarding comprehension of specific items may lead to rewording with the assistance of the PPI group.
GP-facing (questionnaires and patient consent forms)	Issues regarding time taken for consenting patients, any difficulties with comprehension of items and reasons for missing data	Relevant quantitative data will be reported descriptively. Issues will be explored qualitatively with GPs during process evaluation.	Amendments to wording of consent form. Re-costing of incentive payments to practices if necessary, for extra consultation time to allow for consent.
	Issues regarding operation of video-camera to record consultation	Rates of incomplete, unusable or missing recordings will be reported. Issues will be explored qualitatively with GPs during process evaluation, through the researcher’s field notes and through correspondence with practices regarding any setup difficulties.	Review of existing technology and equipment provided, consideration of the need for prompts for GPs to initiate videos
	Issues regarding time taken for questionnaire completion, any difficulties with comprehension of questionnaire items, numbers of questionnaires returned and reasons for missing data	Relevant quantitative data will be reported descriptively. Issues will be explored qualitatively with GPs during process evaluation.	Issues regarding comprehension of specific items may lead to rewording. Re-costing of incentive payments to practices if necessary, for extra consultation time to allow for completion.
Researcher-facing (score sheets and templates)	The ability of members of the research team, to complete OPTION(5) score sheets based on review of video-recorded consultations.	Inter-rater reliability of OPTION(5) scores will be evaluated by calculating the inter-class correlation coefficient on ratings of all videos, aiming for values above 0.75. Comparisons will be made with other studies. The completeness and usability of video-recordings will also be reviewed as described above.	Review of training procedures for OPTION(5) measure if correlation low. Review of appropriateness of the measure in the context of this study if training appears sufficient. Consideration of the need for development of an alternative measure to be piloted ahead of a definitive trial.
	The case note template will be assessed for usability.	Any difficulties with comprehension of items by the research team will be reviewed in team meetings. Any challenges when obtaining the information required on case note review forms, including accessing and interpreting the information from the patient’s medical record, will be reviewed at team meetings. Inter-rater reliability for items on the case note review form will be evaluated by calculating an ICC on 20% of the data.	Re-wording and re-formatting of the template. Review of the qualifications required by the research team e.g. clinical academics only and level of qualification if so. Consideration of time frame for data collection, e.g. is 28 days sufficient to allow the notes to ‘settle’ and to capture all relevant documentation.

The study will assess the appropriateness of data collection procedures for use in the intended participant population as well as for the purposes of evaluating the intervention. Field notes from the research team, process evaluation interviews with participants and research meeting notes will guide an assessment of the appropriateness of measures. Participants’ ability to complete each patient-facing or GP-facing measure will be reviewed.

Assessments of the volume of data collection will establish whether it is appropriate to scale-up in a larger trial. This will include the time taken for field visits, data input and independent checking of the trial database. Overall completeness of data will be established, along with the impact of this on the potential usability of data to evaluate the effectiveness of the intervention.

#### Evaluation of outcome measures

Table 4 shows the data to be collected from patients, GPs and practices for comparability of outcome measures between randomised groups, along with methods of analysis. Analysis will be of observed data only (no statistical methods for addressing missing outcome data, such as multiple imputation, will be used). However, the study will report on reasons for missing outcome data. Any between-group differences, indicating bias, will be evaluated. These evaluations will inform the methods of analysis of both dependent and explanatory outcome variables in the future trial, as well as a review of cost and resource implications. Examples of potential reasons for missing data include loss to follow-up, participants’ completion of questionnaires but non-attendance at the Index consultation, participants attending the consultation but not providing data on questionnaires, data lost or unavailable for other reasons, participants no longer able to experience post-consultation outcomes for example because they have died.

**Table 4:** Evaluation of outcome measure processes

Data	Timing of data collection	Source of data	Type and total possible number of participants providing data	Type of data	Method of analysis
**Baseline**					
Practice characteristics (list size, location, deprivation)	Prior to randomisation	Practice and Association of Public Health Observatories website	6 practices	Categorical, nominal/ordinal.	Frequencies, to report data descriptively. Logistic hierarchical modelling to estimate between group differences^a^ (random effect on cluster, adjustment for practice location).
Patient age, gender, ethnicity, self-reported health status	Prior to index consultation	Pre-consultation postal questionnaire	180 patients	Categorical, nominal/ordinal.	Frequencies, to report data descriptively. Logistic hierarchical modelling to estimate between group differences (random effect on cluster, adjustment for practice location).
Patient deprivation data from patient postcodes	Following return of patient pre-consultation questionnaires and consent forms	Practice records mapped to the Index of Multiple Deprivation	180 patients	Continuous (IMD scale)	Mean and standard deviation, to report data descriptively. Linear hierarchical modelling to estimate between group differences^a^ (random effect on cluster, adjustment for practice location).
GP age, gender, ethnicity, time since qualification	Prior to index consultation	GP practices and General Medical Council GP registry	18 GPs	Categorical, nominal/ordinal	Frequencies, to report data descriptively. Logistic hierarchical modelling to estimate between group differences^a^ (random effect on cluster, adjustment for practice location).
Patients’ preferences for involvement in decision-making.	Prior to index consultation	Patient pre-consultation postal questionnaire	180 patients (90 per arm)	Ordinal (6 point Likert scale).	Frequencies, to report data descriptively. Logistic hierarchical modelling to estimate between group differences^a^ (random effect on cluster, adjustment for practice location).
**Clinical outcomes**					
Putative primary outcome					
Ratings of shared decision-making during the consultation from an observer perspective.	During data analysis	Assessment of video’d consultations by two trained researchers using the OPTION(5) score ([18] [19];)	18 GPs, 180 patients (9 GPs and 90 patients per arm)	Continuous (OPTION score 0-100%)	Mean and standard deviation, to report data descriptively. Linear hierarchical modelling to estimate between group differences* (random effect on cluster, adjustment for practice location).
Additional outcomes					
Patient-reported rating of involvement in decision-making about their healthcare	Immediately following the index consultation	Patient post-consultation questionnaire—using collaboRATE score ([18] [19];)	180 patients (90 per arm)	Continuous (collaboRATE score 0–100%)	Mean and standard deviation, to report data descriptively. Linear hierarchical modelling to estimate between group differences^a^ (random effect on cluster, adjustment for practice location). Patient and GP scores compared using logistic regression modelling (patient scores as outcome, GP scores as explanatory variable).
Patient-reported rating of feeling satisfied with the healthcare received	Immediately following the index consultation	Patient post-consultation questionnaire	180 patients (90 per arm)	Categorical, ordinal (3 point Likert scale)	Frequencies, to report data descriptively. Logistic hierarchical modelling to estimate between group differences^a^ (random effect on cluster, adjustment for practice location).
Patient-reported rating of having trust in the GP they saw				Categorical, ordinal (3 point Likert scale)	Frequencies, to report data descriptively. Logistic hierarchical modelling to estimate between group differences^a^ (random effect on cluster, adjustment for practice location).
Patient-reported rating of enablement				Discrete (PEI score 0–12)	Frequencies, to report data descriptively. Logistic hierarchical modelling to estimate between group differences^a^ (random effect on cluster, adjustment for practice location).
GP-reported rating of their involvement of the patient in decision-making about their healthcare	Immediately following the index consultation, after confirming patient consent for each aspect of data collection.	GP questionnaire using adapted collaboRATE ([18] [19];)	18 GPs (9 per arm)	Continuous (collaboRATE score 0-100%)	Mean and standard deviation, to report data descriptively. Linear hierarchical modelling to estimate between group differences^a^ (random effect on cluster, adjustment for practice location). Patient and GP scores compared using logistic regression modelling (patient scores as outcome, GP scores as explanatory variable).
Patient contacts in a 28-day period following the index consultation, including the nature of contact with the GP surgery, the hospital admissions, A&E attendances. If patient moved away within 28 days (i.e. lost to follow up)	Approximately 12 weeks after index consultation (to allow time for contacts to be recorded in the notes)	Case note review by two researchers	180 patients, (90 per arm)	Count	Median and range, to report data descriptively. Poisson hierarchical modelling to estimate between group differences^a^ (random effect on cluster, adjustment for practice location).
Deaths within a seven day period following the index consultation; death within 28 days (i.e. did not have full study follow-up).	Approximately 12 weeks after index consultation (to allow time for contacts to be recorded in the notes)	Case note review by two researchers	180 patients, (90 per arm)	Count	Median and range, to report data descriptively. Poisson hierarchical modelling to estimate between group differences^a^ (random effect on cluster, adjustment for practice location).
Documented decision outcomes from the index consultation, e.g. starting/stopping/changing medication, referrals and investigations	Approximately 12 weeks after index consultation (to allow time for contacts to be recorded in the notes)	Case note review by two researchers	180 patients, (90 per arm)	Binary (yes/no) variables for each type of change	Frequencies, to report data descriptively. Logistic hierarchical modelling to estimate between group differences^a^ (random effect on cluster, adjustment for practice location).
Process evaluation					
Participant experiences of the intervention, participants experiences of the study	Following receipt of participant post-consultation questionnaires and consent forms	Interviews with the participants from practices assigned to the intervention	9 GPs, 15 patients	Audio-recordings for qualitative analysis	Both deductive and inductive approaches to thematic analysis

^a^Between group differences will be reported using the appropriate outcome metric with 95% confidence intervals; no p-values will be reported in this feasibility study

Using an ‘intention to treat’ approach, participants will be analysed according to their randomised allocation irrespective of intervention actually received. However, all participants will be considered adherent by definition of the protocol. There is no mechanism by which participants in the control group could receive the intervention.

An intra-cluster correlation coefficient (ICC) will be calculated within each arm (intervention and control) for academic purposes; however, this will not be used to calculate a sample size for the future definitive trial; an estimated ICC, calculated from a large relevant routine dataset, will instead be sought [40].

The appropriateness of the outcome measures for use in a future, definitive trial of the intervention will be evaluated. The consistency with which certain outcome measures (e.g. OPTION(5), CollaboRATE, and those items taken from the GPPS survey) perform with the study population, when compared with previous performance in existing literature, will be reviewed. Findings will also be considered in the context of the internal consistency of performance of these measures within the participant sample. Consideration will be given as to whether the outcome measures chosen are sensitive to the effects of the intervention in the context of older people with multimorbidity in primary care, or whether there is a need for development of new measures.

#### Evaluation of resources and capacity to manage the study and implement the intervention

A cost-effectiveness framework for a subsequent definitive trial will be developed by identifying, measuring and valuing the resources used to deliver the intervention. For example, the time required to identify ten consecutive eligible patients per GP will contribute to informing the time required for intervention delivery in the future trial. Data collection processes will be tested with consideration of estimations of the size and range of cost components, along with reporting of the resource use categories prone to missing data. For example, the acceptability of administering questionnaires will be analysed by reporting missing values and presenting mean scores and 95% confidence intervals.

Patient safety data will be described numerically in both intervention and control practices (no hypothesis testing), e.g. hospital admissions, attendances at A&E and deaths within 7 days of the index consultation.

#### Preliminary evaluation of participant responses to the intervention

The intended sample size is sufficient to estimate standard deviations in our intended primary outcome, ‘ratings of shared decision-making during the consultation from an observer perspective’, for the intervention and control group. This outcome data will be obtained from OPTION(5) ratings of video-recorded consultations. No formal power calculations will be carried out and hypothesis testing will not be appropriate [47].

The observer OPTION(5) scores (Elwyn 2014) and the patient and GP ratings of shared decision-making, based on collaboRATE (Elwyn 2014), will be analysed using hierarchical linear modelling with a random effect on cluster. Patient and GP collaboRATE scores will be compared using logistic regression; modelling patient scores as the outcome and GP scores as the explanatory variable, providing preliminary data on whether patient and GP perceptions are congruent.

Patients vary in their pre-existing preferences for involvement in decision-making about their healthcare (Butterworth 2013 [50];). A secondary analysis with adjustment for patients’ reported preference for shared decision-making will provide a preliminary indication as to whether patients’ pre-existing preferences influence the effect of the intervention.

The participant population will be ‘all randomised’ and the analysis strategy will be ‘intention to treat’. There are no plans for interim analysis.

### Process evaluation using qualitative methods

A qualitative interview study, embedded within the feasibility trial with data integration.

Fifteen patients from intervention practices will be sampled and twice this number will be approached. Patients will be selected purposively and iteratively, using questionnaire data to ensure heterogeneity by age, sociodemographic characteristics, health status and preference for shared decision-making (Coyne 2008). Patients will be contacted by telephone to arrange an interview. All GPs in the intervention group will be invited by email. The sample size will be reviewed when assessing thematic saturation [22].

Following receipt of post-consultation participant questionnaires, semi-structured interviews aided by a topic guide will be digitally audio recorded, transcribed and supported by field notes. Participants will be encouraged to discuss their own ideas in order to collect fresh data without the influence of the researcher (Britten et al. 1995). Data will be collected regarding:

Participant experiences of the intervention

- Acceptability of the intervention
- Influences for intervention implementation and integration into normal practice
- Whether participants perceive the intervention to be effective

Participant experiences of the study

- How study participation was experienced
- Whether study participation induced behaviour change not prescribed by the intervention

Audio recordings will be coded using Nvivo computer software (Mills, Bonner and Francis 2006). Data analysis will take a sequential-explanatory approach, combining both deductive and inductive methods to investigate whether the theory developed earlier in the project holds, but also being open to new data and emerging themes [31]. The concept of inductive thematic saturation [21] (Maykut and Morehouse 1994) will be applied and negative cases will be actively sought [44]. The consistency with which the coding is applied will be assessed on a fifth of the data [29]. Analytic meetings will be held with the research team to discuss validity. Data will be interpreted within the context of possible cause–effect pathways of the intervention.

### Criteria for progression to a future definitive randomised controlled trial of the intervention

Study processes are designed to be iterative, formative and adaptive [6]. However, there are certain criteria on which progression to a future, larger trial of intervention effectiveness is dependent. These are:

- Recruitment
  - > 80% of general practices approached (i.e. 5/6)
  - > 60% of GPs approached per practice (i.e. 2/3)
  - > 50% of patients approached per practice (i.e. 20/30)
- Collection of outcomes
  - > 60% of patient-reported outcomes (questionnaire response rate)
  - > 70% of patient questionnaire respondents providing written consent to case-note review
  - > 70% of video recordings useable for observer ratings

Loss to follow up is expected to be minimal and is therefore not included as a ‘stopping criteria’.

The Chief Investigator, the Trial Steering Committee and the sponsor have the authority to stop or advise modifications to the feasibility trial.

### Data management, trial monitoring and participant safety

The Data Management Plan and Data Privacy Impact Assessment are included in the Appendix. Monitoring will be carried out by the Trial Steering Committee in conjunction with the Chief Investigator and the supervisory team. Prospective, planned deviations or waivers to the protocol are not allowed under the UK regulations on Clinical Trials and must not be used. Accidental protocol deviations will be adequately documented, reported to the Chief Investigator, sponsor and ethics committee as indicated.

### Indemnity

Both University of Exeter indemnity arrangements and National Health Service indemnity apply.

### Dissemination

Findings will be published in a peer-reviewed journal, disseminated to participants via GP practices and presented at relevant local and national conferences. The international guidelines from the International Committee of Medical Journal Editors will be applied.

The University of Exeter has a dedicated Research and Knowledge Transfer team to identify, evaluate and commercialise any Intellectual Property resulting from this research.

## Discussion

Shared decision-making during general practice consultations for older people with multimorbidity is currently under-researched and poorly understood [8]. However, emerging clinical guidelines for the management of multimorbidity have advised on a personalised, patient-centred approach when balancing the risks and benefits of treatment; to reduce treatment burden, and to focus on quality of life as well as on specific disease control [33, 34]. The Royal College of General Practitioner, together with NHS England, have a current focus on shared decision-making as a core component of a training programme to deliver personalised care to patients in England [35, 41].

### Comparisons with existing literature

There is currently no firm evidence about the most effective types of intervention for increasing healthcare professionals’ adoption of shared decision-making; however, studies advocate targeting patients and practitioners together [24]. A review of three randomised controlled trials of interventions to improve older patients’ involvement in primary care consultations [50] suggested using face-to-face sessions to encourage practitioner behaviour change along with a written support tool for patients. The review authors advised evaluation using randomised controlled trials, using objective health outcomes over time, a valid assessment from patient, GP and observer perspectives and to correct results for patients’ reported preferences for involvement. A more recent review [8] suggested that transparency in intervention design, testing and evaluation is required to inform the evidence base.

### Strengths and limitations of the study

The systematic development, testing and evaluation of an intervention in this field of research is warranted and timely.

Resources for this doctoral fellowship study are limited, as is researcher capacity, and therefore the participant sample is from one county in the UK only. The patient population in Devon is predominantly of white ethnicity. Consideration will be given to targeting patients of black and other ethnic minorities; however, in some rural areas of Devon, numbers are expected to be limited. Devon also has a higher proportion of older people when compared with the rest of the UK. This may give a false impression of the feasibility of patient recruitment and consideration will be given to this when reporting generalisability of findings. However, GP practices in inner city Plymouth are being targeted with the aim of accessing areas of social deprivation, as well as ethnic diversity.

The findings from this study will inform the decision to progress to a full-sized randomised controlled trial. Quantitative feasibility measures are complimented by qualitative process evaluation, enabling the exploration of both patient and GP participant perceptions of study processes, and to gain insight into barriers and facilitators for successful implementation of the intervention.

If found to be feasible, the future trial design will be adapted to take account of any challenges encountered in this study, to optimise trial processes including recruitment and data collection. Certain challenges are expected. For example, UK GPs, who are currently under considerable workload pressure, may be difficult to recruit. Incentivisation to participate will be used and process evaluation will enable an understanding of whether this was successful. It is also expected that certain vulnerable patient groups, such as those with dementia and those suffering a terminal illness, will be excluded. Consideration will be given as to how vulnerable patients, to whom personalised care is particularly important, could be included in a future trial. For example, the involvement of third-party carers during the consultation will be explored.

The study informs the choice of appropriate good-quality outcome measures for a future trial. The feasibility study is not powered to evaluate intervention effect; however, it is designed to avoid the problem of wasted time and resources which might otherwise occur if data collection and evaluation processes were not tested.

This project seeks to fill the current gap in the literature, to inform clinicians and policy makers in their provision of good-quality patient-centred care of older people with multimorbidity. The research team are well-placed within both clinical and academic primary care to carry out the work, and they have the skills and experience required to complete the proposed study.

### Roles and responsibilities of trial management groups and individuals

#### The project planning group

Key contributors include two representatives of the patient and public involvement (PPI) group; two practicing GPs; a clinical commissioner; two professors in the field of Primary Care research; a professor of Clinical Communication with qualitative research expertise; a professor in Psychology Applied to Health with experience of Intervention Mapping; members of the Exeter Clinical Trials Unit; a trials statistician; a post-doctoral researcher, with expertise in PPI; international experts in the fields of shared decision-making, multimorbidity, and intervention mapping approaches.

#### Trial steering committee

The Trial Steering Committee (TSC) oversees the project milestones, addresses any barriers to study completion and any reasons why the study should not continue. The TSC ensure that the conduct of the study safeguards the safety, rights and wellbeing of participants. The TSC absorbs the role of Data Management Ethics Committee.

#### Sponsors representative

Mrs Pam Baxter, Senior Research Governance Officer, University of Exeter, Lafrowda House, St Germans Road, Exeter, Devon, EX4 6TL. p.r.baxter2@exeter.ac.uk. 01392 723588.

#### Patient and public involvement

The National Institute for Health Research (NIHR) Collaboration for Leadership in Applied Health Research and Care, South West Peninsula (PenCLAHRC), assisted with the identification of a group of eight older members of the public with varying, ages, sex, degrees of morbidity and health service experiences. Members of the group remain involved in prioritising and designing intervention and study materials. They provide a lay perspective on emerging findings.

## Supplementary information

**Additional file 1: Appendix 1.** Data management plan and data privacy impact assessment.

## Data Availability

Not applicable—protocol only.
