# Supplementary material for: Randomised feasibility trial and embedded qualitative process evaluation of a new intervention to facilitate the involvement of older patients with multimorbidity in decision-making about their healthcare during general practice consultations: the VOLITION study protocol
Source: Pilot Feasibility Stud. 2020 Oct 26;6:161. doi: 10.1186/s40814-020-00699-7 (PMC7586675; doi:10.1186/s40814-020-00699-7)
Supplement: Supplementary file 1 — Additional file 1: Appendix 1. Data management plan and data privacy impact assessment. [file 40814_2020_699_MOESM1_ESM.docx]

1. Appendix 1. Data management plan and data privacy impact assessment

# Data management plan

If you are re-using existing data, what licences or terms of use will you have to comply with?

This doctoral research project will generate new Intellectual Property (IP) in the form of an intervention to facilitate the involvement of older patients with multimorbidity in decision-making about their healthcare when visiting a GP. The feasibility trial will inform the development of a future definitive fully powered randomised controlled trial. New data will be created. Data collection tools and source documents are summarised in Table 1. We will not be re-using existing data.

How will new data build on and relate to existing data?

The intervention will include an element of GP education/training and a paper-based patient support tool. The intervention builds on existing IP held by previous researchers and new knowledge will become available as the project develops. Copyright will be included in protocols, questionnaires, leaflets etc. Data Rights will be produced for data, and feedback generated from research as well as Copyright produced on the outcomes of the research.

What types of new data will you create and in what format?

See Table 1.

New materials (e.g. written reports, papers) will be disseminated widely through peer-reviewed publications; respected open access research journals; through various research, clinical, educational and third sector networks; and at seminars and conferences.

Can you estimate the size of the data you will create?

Please refer to Table 1 for the type and possible number of participants providing data.

What methods will you use to capture your data and how will these ensure that your data are high quality?

Patient questionnaires have been constructed from elements of validated patient surveys, including the General Practice Patient Survey (Campbell 2019) and a measure of patient empowerment (McAllister 2012).

Two observers will rate GP consultations using the validated OPTION5 scale (Elwyn 2017). Inter-rater reliability will be evaluated.

A case note review template has been constructed for use by two researchers when reviewing patient notes to ensure accuracy of data capture.

Two researchers will carry out thematic analysis of transcription data for process evaluation interviews and coding will be compared and discussed to ensure reliability of emerging themes.

### Documentation and description

What contextual information is needed for you or someone else to understand your data?

An ‘Intervention Mapping’ approach has been used in the development phase of this project and both theory and evidence have been brought together to describe the context of this intervention. This will be presented alongside the feasibility trial methodology and results data in the study report.

How will you capture contextual information?

As above. We will also capture contextual information during process evaluation interviews with participants.

Will you use any metadata standards?

N/A

### Data protection

Where will you store your data and how will you ensure that they are backed up? Will you use University-managed data storage or will you need to set up your own back-up procedures?

We will use University-managed data storage. Any personal information will be held securely on an encrypted computer drive in a locked university office. Only named researchers will have access to this data. Any paper based information will be held in a locked filing cabinet in the research team office.

How will you secure your data? What methods will you use to restrict access to your sensitive data? Will you encrypt hardware when working off campus?

All investigators and trial site staff must comply with the requirements of the Data Protection Act 1998 with regards to the collection, storage, processing and disclosure of personal information and will uphold the Act’s core principles. The Chief Investigator is the data custodian; a GP and doctoral research fellow, who has an appropriate professional background and an experienced and professional supervisory team.

Each participant will be assigned a research number and data will be stored without the subject’s name or address. Participant data and the linkage code to their research number will be stored in separate locations in locked University filing cabinets and as encrypted files on a University computer. Names and participant details will not be passed on to any third parties and no named individuals will be included in the write up of the results. The only time personal information would be passed on would be if we considered there to be a risk of serious harm to a research participant, and normally this would occur after discussion with the participant concerned. Participant personal data will be anonymised for analysis. In addition, researchers will be bound by any confidentiality requirements unique to individual practices.

Data will be analysed by named members of the research team - primarily the main applicant, their supervisory team, and a trials statistician (Dr Fiona Warren, University of Exeter medical school). Researchers will be bound by any confidentiality requirements unique to individual practices.

How will you protect your research participants? Will you obtain informed consent for data retention and sharing? How will you anonymise data to safeguard the privacy of your participants?

Potential patient participants will be identified using an automated algorithm via the practice computer system resulting in a ‘flag’ on their notes visible to reception staff. Only practice staff will view identifiable personal information of patients flagged by the algorithm. This will be in order to send participant information packs in the post to all potential participants (those aged 65 and older with multiple long-term health problems) at participating practices. When flagged potential participants subsequently attend the waiting room they will be consented for data collection as per the study protocol. Patients will be provided with transparent information about the reasons for contact at the time of receiving the patient information pack in the post.

Personal contact details will be requested by the research team on patient questionnaires in order to contact for telephone interviews. Patients will provide this information with informed (participant information leaflet and discussion with GP), written consent.

We plan to video-record the consultation between patient and GP. Written consent from both parties will be obtained for this, video-recordings will be stored on an encrypted hard drive and files will be labelled with anonymised identifiers.

The clinical record will only be viewed where participants have given consent for this to take place. No patient identifiable data will be removed from the practice. Any data that is collected will be anonymised and saved on an encrypted hard drive. Case note reviews will only take place within the GP surgeries themselves; therefore, there will be no need to remove patient records from the surgeries. Data extracted will be encrypted and will not include names of individuals. Any temporary storage of data on mobile devices (laptops, memory sticks, emails etc.) will be carried out on encrypted devices in accord with NHS security requirements.

Audio-recorded and video-recorded data will be encrypted, stored and transported as per the case note data. Audio-recorded data sent for external transcription will be destroyed within thirty days. Interview transcribers will have signed confidentiality agreements. All transcriptions will be anonymised with any personal details being deleted or replaced by pseudonyms. We may publish anonymised direct quotes in our qualitative interview work; however, we will choose these carefully so as not to include potentially identifiable information.

### Retention and preservation

Which subsets of your data will you keep at the end of your project? Will you retain anonymised versions but destroy personal data and identification keys? Will you retain all of the raw data or is a processed version more suitable to preserve? Do you need to keep all intermediary files or would you only need to refer back to input files or a final version?

Personal data and identification keys will be stored for 6 months after the end of the study.

The raw data (audio-recordings) will be destroyed after 6 months; however, transcriptions generated from the study will be anonymised and stored for 5 years.

How will you prepare your data for long-term preservation? Are you able to convert your data to open file formats? What contextual information do you need to retain so that your data remain understandable and usable?

Data will be stored in a locked University filing cabinet and on an encryted drive on a University computer.

Where will you archive your data to ensure that they are preserved and sustained for several years after your project ends? Will you submit your data to a specialist data repository/centre and if so, have you consulted them about your requirements?

The data will be archived at the University of Exeter medical school.

How big will your final dataset be and will there be any costs associated with archiving them, such as data deposit charges?

No costs will apply. The data set will be relatively small.

### Data sharing

Can you demonstrate that you'll plan ahead to maximise data sharing? For example, will you only share a subset of the data where informed consent was granted for data sharing?

We do not plan to share the raw data from this study and will not consent participants to do so. We plan to disseminate the results only.

Are there any reasons why you would not be able to share some of your data? Would they be covered by data protection legislation, licence restrictions, or contractual confidentiality clauses? Are there ethical reasons why data should not be released?

We do not see any reasons why the anonymised results from this study should not be shared.

With regard to the intervention components to be tested:

The University of Exeter (UoE) has a dedicated Research and Knowledge Transfer (RKT) team to identify, evaluate and commercialise IP resulting from research. All new IP will vest in the relevant party that creates it, with a licence to the UoE for the purposes of research, teaching and publication. The IP generated will be recognised, captured, and managed by the research team with the support of the RKT team. The chief investigator and supervisors will hold the IP associated with materials developed from the study. No direct action is needed to register Copyright for the outcomes of this project, but we will work with the IP Manager at the University of Exeter to ensure all proper procedures are taken. The IP generated will be recognised, captured, and managed by the chief investigator (supported by the supervisory team and the University’s RKT team). Although new IP generated from the resultant intervention will be owned by the UoE, it will be made freely available to NHS commissioners and providers.

Although not expected, should new IP have a commercial potential, the researchers will notify the UoE’s IP & Commercialisation team who manage exploitation. Commercialisation would comply with requirements of the funding body and will not affect the University’s right to publish. We do not envisage any potential regulatory barriers to new IP being utilised in the NHS. There should be no regulatory hurdles to the outcomes of this research as regulatory bodies will be forewarned of the proposal and its potential outputs. As key stakeholders will be fully engaged from the outset of the research it will be a matter of disseminating knowledge and adoption of best practice to a wider audience of healthcare services. To mitigate against any unforeseen hurdles that may present, the university’s RKT team also has a direct link with the South West Academic Health Science Network (AHSN) and will engage closely with the AHSN in assisting with the dissemination and adoption of the approaches that will result from the research.

When will you share your data? Will data be made available upon first publication of findings or within a limited period after the end of the project? Do you need to delay publication to allow for commercialisation or patent applications? Will you embargo your data to allow for a limited period of exclusive use?

Data will be made available on first publication of findings. There will be no reason to delay publication. The feasibility trial will inform the development of a fully powered randomised controlled trial of the intervention.

How will you disseminate your research? Will you include a data access statement in published articles? Does your chosen method of data preservation provide a persistent URL such as a Digital Object Identifier? What licences will you assign to your data?

The findings will be published in a peer review journal, disseminated to participants via GP practices, and presented at relevant local and national conferences. There will be no need to assign licences (see notes re IP above).

Data protection impact assessment

What do you require this personal data for? What is the purpose of using the personal data?

Potential participants will be identified by the practice administration team, using an algorithm to enable a computerised search of GP records. Following identification of potential participants using a computer algorithm, practice administration staff will use identifiable information to send patient invitations and information sheets in the post. Those patients wishing to participate will return their reply sheets to the researcher. Reply sheets will contain participant contact details and the name and practice of their registered GP. Participants will also supply their age, and the number and type of long-term health problem(s) they have.

Further personal information will not be required by the researcher but the registered GP will be asked to screen the medical records for any reason why the participant should be excluded. Any personal information received on reply sheets will be anonymised by the researcher and participants will subsequently be identified by number only. Reply sheets will be stored securely in a locked University filing cabinet in a locked office.

How are you making people aware of how their personal data is being used? Do you need to update your privacy notice?

The participant information sheet will provide this information:

‘Who will have access to my information?’

Due to recent regulatory changes in the way that data is processed (General Data Protection Regulation 2018 and the Data Protection Act 2018), the University of Exeter’s lawful basis to process personal data for the purposes of carrying out research is termed as a ‘task in the public interest’. The University will endeavour to be transparent about its processing of your personal data and this information sheet should provide a clear explanation of this. If you do have any queries about the University’s processing of your personal data that cannot be resolved by the research team, further information may be obtained from the University’s Data Protection Officer by emailing [dataprotection@exeter.ac.uk](mailto:dataprotection@exeter.ac.uk) or at [www.exeter.ac.uk/dataprotection](http://www.exeter.ac.uk/dataprotection). If you have any concerns about how the data is controlled and managed for this study, then you can also contact the Sponsor Representative, Pam Baxter, Senior Research Governance Officer, whose details are at the end of the information sheet.

For the purposes of this study, we will also use consent to protect your confidentiality and provide you with choice in your participation. All information collected in this study will be kept strictly confidential and stored either on an encrypted password protected computer, or in a locked cabinet at the University, which can only be accessed by the researcher (and research supervisors). You will be allocated a unique participant number, which will ensure the information from your questionnaires, video observations, case note review and interview will be protected and cannot be identified by anyone else. Any personally identifiable information will be stored separately and securely from information obtained from the research, for example your contact information required to arrange the interview, and will be securely destroyed after 5 years. Archived files will be stored in a locked cabinet at the University.

Your rights to access, change or move your information are limited, as we need to manage your information in specific ways in order for the research to be reliable and accurate. If you withdraw from the study, we will keep the information about you that we have already obtained. To safeguard your rights, we will use the minimum personally-identifiable information possible.

Which conditions for processing apply for your project? For Special Categories please ensure you select at least one from Section 1 and one from Section 2 below. Please select all that apply and provide any additional details.

Section 1: Conditions for personal data

- The data subject has given consent to the processing (please provide the consent wording and where it is stored)
- Contractual necessity (please confirm which contract this relates to)
- Compliance with any legal obligation (please document which legal obligation)
- To protect the vital interests of the data subject (please provide details)
- Functions of a public nature or task in the public interest (please provide details)
- Legitimate interest of the Data Controller (please provide details of legitimate interest)

Section 2: Conditions for special categories data

- The data subject has given explicit consent to the processing
- Necessary so that you can comply with employment law
- To protect the vital interests of the data subject or *other person*
- The processing is carried out as part of the legitimate activities of a not-for-profit organisation
- The individual has deliberately made the information public
- The processing is necessary in relation to legal rights
- The processing is necessary for administering justice or for exercising statutory or governmental functions
- The processing is necessary for medical purposes
- The processing is necessary for monitoring equality of opportunity

The data subject has given consent to the processing:

‘I understand that data collected during the study may be looked at by individuals from the University of Exeter Medical School, from regulatory authorities, or from the GP practice. Where it is relevant to my taking part in this research I give permission for these individuals to have access to my data.’

Consent forms will be stored in a locked University filing cabinet.

Is all the personal data you are using necessary? Are you collecting enough to carry out the work, is there any you could do without to limit the risks to the individuals?

The personal data collected is the minimum necessary to be able to determine inclusion criteria and to contact the patient to arrange an interview. It will also enable us to contact the GP to determine whether the patient is suitable for the study (to consider exclusion criteria).

How are you ensuring that personal data obtained from individuals or other organisations is accurate? How will you keep it updated?

Initial patient contact will be made via the GP practice database, which is deemed to be a fairly reliable source, and participants will subsequently be asked for their name and contact information on reply sheets.

How long will you keep the data and how will you dispose of it? Are the retention periods on the University Retention Schedule?

Personal data will be kept for no longer than 6 months.

Where will the data be stored? If storage is in the cloud, where is the physical server? Will you need to transfer the data outside the EEA? If yes, how will you ensure adequate protection?

Personal data on paperwork will be stored in a locked University filing cabinet.

Will you be able to meet all the Data Subject Rights? Can you provide copies of data if requested? Are you able to fully delete the data (not just archive)?

Copies of personal data and anonymised study data will be available on participant request. It will be possible to completely destroy personal data.

Please briefly document below any risks with the use of personal data and how you will control such risks. Include technical controls (IT security, encryption etc.), physical controls (location, locked room etc.), personnel controls (training, access control etc.) and procedural controls (contract, polices etc.).

The main risk with personal data is the potential for breach of confidentiality.

Any personal information will be held securely on an encrypted computer drive in a locked university office. Only named researchers will have access to this data. Any paper based information will be held in a locked filing cabinet in the research team office.

Only staff attached to the practices will be involved in accessing patient records. Audio-recorded data will be transcribed and encrypted and will not include names of individuals. Video-recorded data will be transcribed. Any temporary storage of data on mobile devices (laptops, memory sticks, emails etc.) will be carried out on encrypted devices in accordance with NHS security requirements. Audio-recorded data sent for external transcription will be destroyed within thirty days.

All personal data obtained from patients, GPs or staff (e.g. names, addresses, contact details, personal information) for both the purposes of recruitment and data collection, will remain confidential and held in accordance with the Data Protection Act.

Each participant will be assigned a research number and data will be stored without the subject’s name or address. Names and participant details will not be passed on to any third parties and no named individuals will be included in the write up of the results. The only time personal information would be passed on would be if we considered there to be a risk of serious harm to a research participant, and normally this would occur after discussion with the participant concerned. Participant personal data will be anonymized for analysis. In addition, researchers will be bound by any confidentiality requirements unique to individual practices.

Interview transcribers will have signed confidentiality agreements. All transcriptions will be anonymised with any personal details being deleted or replaced by pseudonyms.

Only named researchers will have access to personal data, with informed written consent from the participant.

The participant information sheet will inform participants about who may have access to their medical records and trial data, and why.

The named primary researcher will be the lead applicant, a GP and doctoral research fellow, who therefore has an appropriate professional background and an experienced and professional supervisory team.

Data will not be exported outside of the UK.

Data will be held on an encrypted University computer and in a locked filing cabinet in a secure University office. It will be analysed by named members of the research team—primarily the main applicant and their supervisory team. Anonymised data may be reviewed with the patient and public involvement group.

Table 1 Participant inclusion and exclusion criteria

| Participant type | Inclusion criteria | Exclusion criteria |
| --- | --- | --- |
| GPs | Only permanent GPs (partners and salaried) to avoid loss to follow-up. | Locum (sessional) GPs  Trainee GPs  (GPs will not be excluded based on less than full time working) |
| Patients | Patients aged 65 and above with known multimorbidity  The condition should be one of^a^:  angina or long-term heart problem; arthritis or long-term joint problem; asthma or long-term chest problem; blindness or severe visual impairment; cancer in the last five years; deafness or severe hearing impairment; diabetes; epilepsy; high blood pressure; kidney or liver disease; long-term back problem; long-term mental health problem; long-term neurological problem. | Temporary residents and vulnerable patients e.g. those recently bereaved, those with severe mental illness, severe cognitive impairment, end stage disease, communication difficulties e.g. physical impairment caused by a stroke as opposed to language barriers, a learning disability, or those unable to complete questionnaires or interviews for any other reason.  (A minimum time for a condition to be ‘long-term’ will not be specified) |

^a^This list was adapted from the English National General Practice Patient Survey (Campbell 2009). Where dyads of conditions occur within the same organ system, e.g. anxiety and depression, these will only be counted once e.g. mental health problem

Table 2 The VOLITION intervention applications

| Patient support tool* | GP workshop |
| --- | --- |
| Central image | Delivery and facilitation by a GP |
| Illustrates the spectrum of patient preferences for involvement and poses the question “where do you see yourself?” | For the purposes of role-modelling as a means of knowledge transfer |
| Phrases to use during the consultation | Information provision |
| Aimed at facilitating patients to ask for or decline participation in decision-making, accompany the central image. | GP-facilitator provides information regarding the elements of a shared decision-making approach to the consultation, in the context of older patients with multimorbidity. |
| Phrases are matched to the spectrum of patient preferences for involvement. | GP-facilitator delivers new messages about the potential benefits of shared decision-making. GP-facilitator uses a set of one or more meaningful premises and a conclusion to deliver these messages. |
| Messages | GP-facilitator provides positive messages regarding the role of the patient and their preferences within shared decision-making. (This information is designed using evidence from the literature regarding GPs current beliefs around shared decision-making with this patient group.) |
| Inform the patient of their right to ask for involvement in decision-making about their care. | GP facilitator provides information about the importance and relevance of a patient-centred approach to the consultation. |
| Suggest that the patient possesses the capability to state their preferences for involvement to the GP. | Messages delivered by GP-facilitator suggest that the GP possesses the capability to use a shared decision-making approach to the consultation. |
|  | GPs are asked to relay GP-facilitator’s messages to each other, and facilitator clarifies any confusion that appears during this process. |
|  | Role-play |
|  | GPs are first shown an example video-recorded consultation between the GP-facilitator (peer-model) and an actor-patient. GP-facilitator discusses the challenges of facilitating shared decision-making with the patient and how (s)he overcame them. |
|  | GPs take turns as the GP, the patient and the observer to role-play clinical scenarios in threes. |
|  | Individual GPs rehearse and repeat a shared decision-making approach to a role-play consultation, using a new ‘VOLITION’ model, incorporating a patient-centred, holistic approach. |
|  | The role-play ‘patient’ states their preference for involvement in decision-making about their healthcare; their ideas, concerns and expectations; and their preferred data format for decision-related information. In this way the ‘patient’ prompts the GP to use appropriate communication skills to match their shared decision-making preferences. |
|  | Clinical scenarios provide increasingly challenging tasks during role-play, with feedback from peers serving as an indicator of capability to the GP |
|  | GP-facilitator encourages elaboration to augment the information provided in the crib sheet for the case scenario. |
|  | Reflective discussion in threes followed by group feedback |
|  | Discussion of GP’s appropriate response to patient preferences, fundamental priorities and requirements |
|  | Discuss the experience and provide feedback to others. |
|  | GP-facilitator encourages elaboration to augment the information provided in the crib sheet for the case scenario. |
|  | Supporting reference materials |
|  | Handbook containing the VOLITION model and all of the key messages delivered by the GP-facilitator during the workshop, the case scenarios for role-play and space for the individual to write reflective notes. Also available online. |
|  | Online link to the video-recorded consultation between GP-facilitator and actor-patient. |
|  | Patient dialogue during index consultation |
|  | Patient provides a nudge to the GP in the form of a phrase from the patient support tool, informing the GP of their preferences for involvement in decision making about their care. |
|  | Acts as a cue to the GP to adapt their communication skills accordingly. |

^a^The support tool will also be displayed as a poster in the waiting room to prompt recall, as well as being available in leaflet form

Table 3 Evaluation of data collection procedures

| Type of data collection (patient-, GP- or researcher-facing) | Components to be reviewed | Methods of analysis | Potential resultant amendments ahead of a definitive, larger trial |
| --- | --- | --- | --- |
| Patient-facing (questionnaires) |  | All patient-facing data collection forms have been reviewed for their suitability by Patient and Public Involvement (PPI) group | PPI members advised on the wording and format of questionnaires. PPI will provide a lay perspective on interpretation of feasibility findings. |
|  | The feasibility of distribution and collection of patient questionnaires by receptionists. | Researcher’s field notes and follow-up correspondence with practices.  Process evaluation interviews with patients. | Consideration of distribution by another means e.g. in the post (or email) prior to consultation. |
|  | The appropriateness of collecting demographic data by patient self-report on pre-consultation questionnaires, as opposed to through the medical record. | Review of completeness of relevant items on patient questionnaires. Process evaluation interviews to discuss potential reasons for missing data. Correspondence with practices regarding practicalities of accessing patient demographic data with consent. | Consideration of accessing patient baseline data through practice records. |
|  | Whether patient participants require assistance, encouragement or supervision to complete either of the questionnaires. | Researcher’s field notes and process evaluation interviews. | The provision of assistance to patients when completing questionnaires, e.g. providing staff to assist them or allowing more time pre- or post-consultation to allow the patient to seek help from a third party. |
|  | Issues regarding time taken for questionnaire completion, any difficulties with comprehension of questionnaire items, numbers of questionnaires returned and reasons for missing data | Relevant quantitative data will be reported descriptively. Issues will be explored qualitatively with patients during process evaluation. | Time taken for completion may inform decisions surrounding when and how to distribute questionnaires. Issues regarding comprehension of specific items may lead to rewording with the assistance of the PPI group. |
| GP-facing (questionnaires and patient consent forms) | Issues regarding time taken for consenting patients, any difficulties with comprehension of items and reasons for missing data | Relevant quantitative data will be reported descriptively. Issues will be explored qualitatively with GPs during process evaluation. | Amendments to wording of consent form. Re-costing of incentive payments to practices if necessary, for extra consultation time to allow for consent. |
|  | Issues regarding operation of video-camera to record consultation | Rates of incomplete, unusable or missing recordings will be reported. Issues will be explored qualitatively with GPs during process evaluation, through the researcher’s field notes and through correspondence with practices regarding any setup difficulties. | Review of existing technology and equipment provided, consideration of the need for prompts for GPs to initiate videos |
|  | Issues regarding time taken for questionnaire completion, any difficulties with comprehension of questionnaire items, numbers of questionnaires returned and reasons for missing data | Relevant quantitative data will be reported descriptively. Issues will be explored qualitatively with GPs during process evaluation. | Issues regarding comprehension of specific items may lead to rewording. Re-costing of incentive payments to practices if necessary, for extra consultation time to allow for completion. |
| Researcher-facing (score sheets and templates) | The ability of members of the research team, to complete OPTION(5) score sheets based on review of video-recorded consultations. | Inter-rater reliability of OPTION(5) scores will be evaluated by calculating the inter-class correlation coefficient on ratings of all videos, aiming for values above 0.75. Comparisons will be made with other studies.  The completeness and usability of video-recordings will also be reviewed as described above. | Review of training procedures for OPTION(5) measure if correlation low. Review of appropriateness of the measure in the context of this study if training appears sufficient. Consideration of the need for development of an alternative measure to be piloted ahead of a definitive trial. |
|  | The case note template will be assessed for usability. | Any difficulties with comprehension of items by the research team will be reviewed in team meetings.  Any challenges when obtaining the information required on case note review forms, including accessing and interpreting the information from the patient’s medical record, will be reviewed at team meetings.  Inter-rater reliability for items on the case note review form will be evaluated by calculating an ICC on 20% of the data. | Re-wording and re-formatting of the template.  Review of the qualifications required by the research team e.g. clinical academics only and level of qualification if so.  Consideration of time frame for data collection, e.g. is 28 days sufficient to allow the notes to ‘settle’ and to capture all relevant documentation. |

Table 4 Evaluation of outcome measure processes

| Data | Timing of data collection | Source of data | Type and total possible number of participants providing data | Type of data | Method of analysis |
| --- | --- | --- | --- | --- | --- |
| Baseline | | | | | |
| Practice characteristics (list size, location, deprivation) | Prior to randomisation | Practice and Association of Public Health Observatories website | 6 practices | Categorical, nominal/ordinal. | Frequencies, to report data descriptively.  Logistic hierarchical modelling to estimate between group differences^a^ (random effect on cluster, adjustment for practice location). |
| Patient age, gender, ethnicity, self-reported health status | Prior to index consultation | Pre-consultation postal questionnaire | 180 patients | Categorical, nominal/ordinal. | Frequencies, to report data descriptively.  Logistic hierarchical modelling to estimate between group differences (random effect on cluster, adjustment for practice location). |
| Patient deprivation data from patient postcodes | Following return of patient pre-consultation questionnaires and consent forms | Practice records mapped to the Index of Multiple Deprivation | 180 patients | Continuous (IMD scale) | Mean and standard deviation, to report data descriptively.  Linear hierarchical modelling to estimate between group differences^a^ (random effect on cluster, adjustment for practice location). |
| GP age, gender, ethnicity, time since qualification | Prior to index consultation | GP practices and General Medical Council GP registry | 18 GPs | Categorical, nominal/ordinal | Frequencies, to report data descriptively.  Logistic hierarchical modelling to estimate between group differences^a^ (random effect on cluster, adjustment for practice location). |
| Patients’ preferences for involvement in decision-making. | Prior to index consultation | Patient pre-consultation postal questionnaire | 180 patients (90 per arm) | Ordinal (6 point Likert scale). | Frequencies, to report data descriptively.  Logistic hierarchical modelling to estimate between group differences^a^ (random effect on cluster, adjustment for practice location). |
| Clinical outcomes | | | | | |
| Putative primary outcome | | | | | |
| Ratings of shared decision-making during the consultation from an observer perspective. | During data analysis | Assessment of video’d consultations by two trained researchers using the OPTION(5) score (Elwyn 2013) | 18 GPs, 180 patients  (9 GPs and 90 patients per arm) | Continuous (OPTION score 0-100%) | Mean and standard deviation, to report data descriptively.  Linear hierarchical modelling to estimate between group differences* (random effect on cluster, adjustment for practice location). |
| Additional outcomes | | | | | |
| Patient-reported rating of involvement in decision-making about their healthcare | Immediately following the index consultation | Patient post-consultation questionnaire—using collaboRATE score (Elwyn 2013) | 180 patients (90 per arm) | Continuous (collaboRATE score 0–100%) | Mean and standard deviation, to report data descriptively.  Linear hierarchical modelling to estimate between group differences^a^ (random effect on cluster, adjustment for practice location).  Patient and GP scores compared using logistic regression modelling (patient scores as outcome, GP scores as explanatory variable). |
| Patient-reported rating of feeling satisfied with the healthcare received | Immediately following the index consultation | Patient post-consultation questionnaire | 180 patients (90 per arm) | Categorical, ordinal (3 point Likert scale) | Frequencies, to report data descriptively.  Logistic hierarchical modelling to estimate between group differences^a^ (random effect on cluster, adjustment for practice location). |
| Patient-reported rating of having trust in the GP they saw |  |  |  | Categorical, ordinal (3 point Likert scale) | Frequencies, to report data descriptively.  Logistic hierarchical modelling to estimate between group differences^a^ (random effect on cluster, adjustment for practice location). |
| Patient-reported rating of enablement |  |  |  | Discrete (PEI score 0–12) | Frequencies, to report data descriptively.  Logistic hierarchical modelling to estimate between group differences^a^ (random effect on cluster, adjustment for practice location). |
| GP-reported rating of their involvement of the patient in decision-making about their healthcare | Immediately following the index consultation, after confirming patient consent for each aspect of data collection. | GP questionnaire using adapted collaboRATE (Elwyn 2013) | 18 GPs (9 per arm) | Continuous (collaboRATE score 0-100%) | Mean and standard deviation, to report data descriptively.  Linear hierarchical modelling to estimate between group differences^a^ (random effect on cluster, adjustment for practice location).  Patient and GP scores compared using logistic regression modelling (patient scores as outcome, GP scores as explanatory variable). |
| Patient contacts in a 28-day period following the index consultation, including the nature of contact with the GP surgery, the hospital admissions, A&E attendances. If patient moved away within 28 days (i.e. lost to follow up) | Approximately 12 weeks after index consultation (to allow time for contacts to be recorded in the notes) | Case note review by two researchers | 180 patients, (90 per arm) | Count | Median and range, to report data descriptively.  Poisson hierarchical modelling to estimate between group differences^a^ (random effect on cluster, adjustment for practice location). |
| Deaths within a seven day period following the index consultation; death within 28 days (i.e. did not have full study follow-up). | Approximately 12 weeks after index consultation (to allow time for contacts to be recorded in the notes) | Case note review by two researchers | 180 patients, (90 per arm) | Count | Median and range, to report data descriptively.  Poisson hierarchical modelling to estimate between group differences^a^ (random effect on cluster, adjustment for practice location). |
| Documented decision outcomes from the index consultation, e.g. starting/stopping/changing medication, referrals and investigations | Approximately 12 weeks after index consultation (to allow time for contacts to be recorded in the notes) | Case note review by two researchers | 180 patients, (90 per arm) | Binary (yes/no) variables for each type of change | Frequencies, to report data descriptively.  Logistic hierarchical modelling to estimate between group differences^a^ (random effect on cluster, adjustment for practice location). |
| Process evaluation | | | | | |
| Participant experiences of the intervention, participants experiences of the study | Following receipt of participant post-consultation questionnaires and consent forms | Interviews with the participants from practices assigned to the intervention | 9 GPs, 15 patients | Audio-recordings for qualitative analysis | Both deductive and inductive approaches to thematic analysis |

^a^Between group differences will be reported using the appropriate outcome metric with 95% confidence intervals; no p-values will be reported in this feasibility study

Fig. 1 Testing VOLITION: schedule of enrolment, interventions and assessments

Fig. 2 The VOLITION intervention
